# Plant Cell Wall Polysaccharide *O*-Acetyltransferases

**DOI:** 10.3390/plants13162304

**Published:** 2024-08-19

**Authors:** Ruiqin Zhong, Dayong Zhou, Lirong Chen, John P. Rose, Bi-Cheng Wang, Zheng-Hua Ye

**Affiliations:** 1 Department of Plant Biology, University of Georgia, Athens, GA 30602, USA; 2 Department of Biochemistry and Molecular Biology, University of Georgia, Athens, GA 30602, USA

**Keywords:** acetyltransferase, cell wall, mannan, pectin, TBL, xylan, xyloglucan

## Abstract

Plant cell walls are largely composed of polysaccharide polymers, including cellulose, hemicelluloses (xyloglucan, xylan, mannan, and mixed-linkage β-1,3/1,4-glucan), and pectins. Among these cell wall polysaccharides, xyloglucan, xylan, mannan, and pectins are often *O*-acetylated, and polysaccharide *O*-acetylation plays important roles in cell wall assembly and disease resistance. Genetic and biochemical analyses have implicated the involvement of three groups of proteins in plant cell wall polysaccharide *O*-acetylation: trichome birefringence-like (TBL)/domain of unknown function 231 (DUF231), reduced wall acetylation (RWA), and altered xyloglucan 9 (AXY9). Although the exact roles of RWAs and AXY9 are yet to be identified, members of the TBL/DUF231 family have been found to be *O*-acetyltransferases responsible for the *O*-acetylation of xyloglucan, xylan, mannan, and pectins. Here, we provide a comprehensive overview of the occurrence of *O*-acetylated cell wall polysaccharides, the biochemical properties, structural features, and evolution of cell wall polysaccharide *O*-acetyltransferases, and the potential biotechnological applications of manipulations of cell wall polysaccharide acetylation. Further in-depth studies of the biochemical mechanisms of cell wall polysaccharide *O*-acetylation will not only enrich our understanding of cell wall biology, but also have important implications in engineering plants with increased disease resistance and reduced recalcitrance for biofuel production.

## 1. Introduction

Land plants are estimated to produce approximately 56 billion metric tons of fixed carbon via photosynthesis annually, half of which are stored in forest trees [1]. Because wood, which is mainly made of cell wall polymers, constitutes the bulk of the biomass of forest trees, cell wall polymers are considered to be the most abundant biomass and stored carbon by plants. Considering the sheer volume of cell wall polymers produced by plants and the wide-range applications of cell wall polymers in the form of wood and fibers in our daily lives, it is imperative to unravel the biochemical mechanisms controlling the biosynthesis of cell wall polymers. The knowledge gained from such studies will potentially provide molecular and genetic tools to customize the composition of cell wall polymers suited to our diverse end uses.

**Figure 1:**
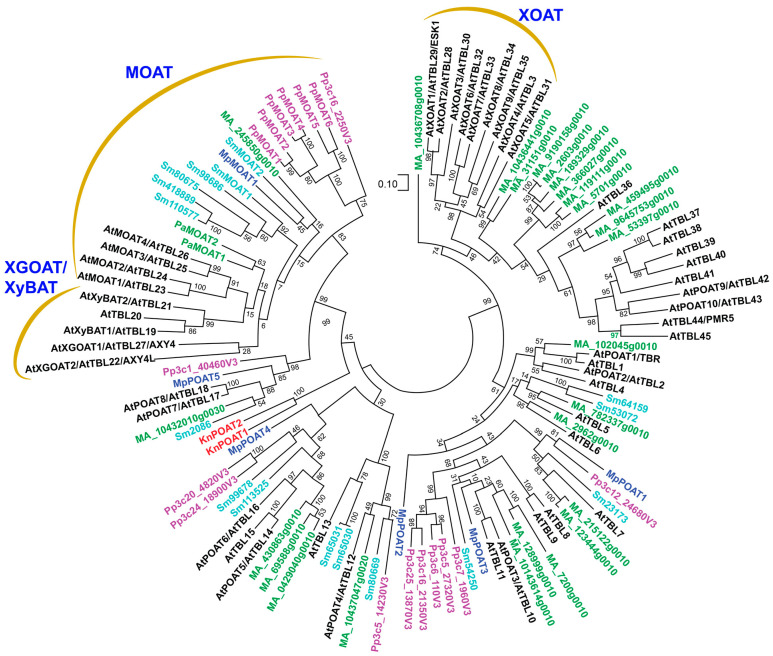
Phylogenetic relationship of TBL members from *Arabidopsis* (At), *Picea abies* (Pa/Ma), *Selaginella moellendorffii* (Sm), *Physcomitrium patens* (Pp), *Marchantia polymorpha* (Mp), and *Klebsormidium nitens* (Kn). The phylogenetic tree was constructed using MEGA11 software with the maximum likelihood method. The numbers at the nodes represent bootstrap values as percentages of 1000 replicates and the 0.1 scale denotes 10% change.

Plant cell walls are mainly composed of polysaccharides, including cellulose, hemicelluloses (xyloglucan, xylan, mannan, and mixed-linkage β-1,3/1,4-glucan), and pectins, which are interwoven into a complex structure vital for mechanical support and protection to the plant body. In some specialized cells, such as tracheary elements and fibers, the polyphenolic polymer lignin is produced and impregnated into the cell wall polysaccharide network, conferring increased mechanical strength, rigidity, and hydrophobicity to the cell walls. Among these cell wall polymers, xyloglucan, xylan, mannan, pectins, and lignin are often *O*-acetylated, and their acetyl esterification plays important roles in cell wall structure, disease resistance, and plant development [2,3,4,5]. For example, *Arabidopsis* mutants defective in xylan acetylation exhibit a disorganized secondary wall structure, collapsed xylem vessels, and impaired plant development [6,7,8], and those defective in pectin acetylation show reduced cell expansion and increased disease resistance [9,10,11]. Although enzymes mediating the *O*-acetylation of lignin units have not been identified [12], three groups of Golgi-localized proteins, including trichome birefringence (TBR)-like, (TBL)/domain of unknown function 231 (DUF231), reduced wall acetylation (RWA), and altered xyloglucan 9 (AXY9), have been found to be involved in the *O*-acetylation of plant cell wall polysaccharides. A number of TBL proteins have been demonstrated to be *O*-acetyltransferases catalyzing acetyl transfer onto specific cell wall polysaccharides (Figure 1), whereas RWAs and AXY9 are required for the *O*-acetylation of all cell wall polysaccharides, but their biochemical activities remain elusive. In this review, we discuss the current understanding of plant cell wall polysaccharide *O*-acetyltransferases and highlight the outstanding issues that await investigation.

## 2. Xyloglucan *O*-Acetyltransferases

Xyloglucan, a ubiquitous hemicellulose found in the primary walls of plant cells, consists of a backbone of β-1,4-linked D-glucosyl (Glc) residues that are often substituted at *O*-6 with α-D-xylosyl (Xyl) residues [13]. These Xyl side chains can be decorated at *O*-2 with additional sugar residues, such as β-D-galactose (Gal), β-D-galacturonic acid (GalA), α-L-arabinofuranose (Ara*f*), α-L-arabinopyranose (Ara*p*), or β-D-Xyl, depending on plant species, and the Gal residues may be further substituted at *O*-2 with α-L-fucose (Fuc). Based on the xylosylation pattern, xyloglucan is generally classified into two types: the XXXG type and the XXGG_n_ type. The XXXG type is composed of repeating units of three consecutive xylosylated Glc residues (denoted as X) [14] separated by a single non-xylosylated Glc (denoted as G) and the XXGG_n_ type comprises repeating units of two consecutive xylosylated Glc residues followed by two or more non-xylosylated Glc. The former is present in most vascular plants and hornworts and the latter is predominant in grasses, Solanaceae (e.g., tomato and tobacco), liverworts, and mosses [13].

**Figure 2:**
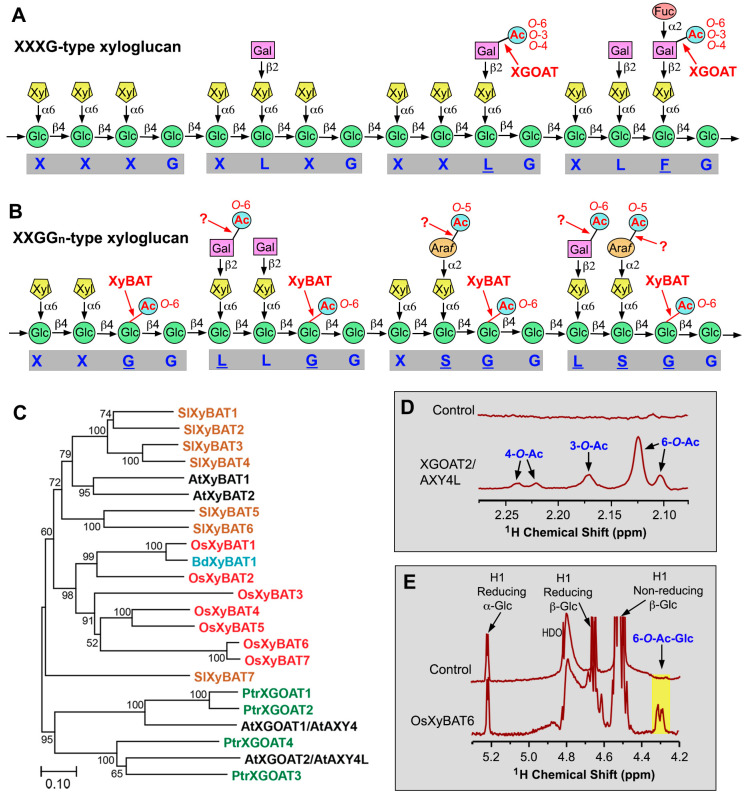
*O*-acetyltransferases mediating *O*-acetylation of xyloglucan. (**A**) Diagram of representative structural motifs of the XXXG-type xyloglucan showing acetyl groups attached to side-chain Gal residues. The letters underneath the diagram denote side chain structures and the underlined letters indicate the presence of *O*-acetyl groups. Question marks indicate enzymes that have not been identified or biochemically verified. (**B**) Diagram of representative structural motifs of the tomato XXGG_n_-type xyloglucan showing acetyl groups attached to side-chain Gal and Ara*f* residues and to backbone Glc residues. The question marks indicate that the corresponding *O*-acetyltransferases have not yet been identified. (**C**) Phylogenetic relationship of biochemically characterized XGOATs and XyBATs from *Arabidopsis* (At), poplar (*Populus trichocarpa*; Ptr), tomato (*Solanum lycopersicum*; Sl), rice (*Oryza sativa*; Os), and *Brachypodium distachyon* (Bd). The phylogenetic tree was constructed using MEGA11 software with the maximum likelihood method. The numbers at the nodes represent bootstrap values as percentages of 1000 replicates and the 0.1 scale denotes 10% change. (**D**) ^1^H-NMR spectra of the acetyl resonance region of unacetylated xyloglucan oligomers (control) and acetylated xyloglucan oligomers catalyzed by XGOAT2/AXY4L showing resonance signals corresponding to acetyl groups attached to *O*-3, *O*-4, and *O*-6 of side-chain Gal residues. See Zhong et al. (2018) [22] for details. (**E**) ^1^H-NMR spectra of unacetylated cellohexaose (control) and acetylated cellohexaose catalyzed by OsXyBAT6 showing resonance signals corresponding to 6-*O*-acetylated backbone Glc residues (highlighted in yellow). See Zhong et al. (2020) [26] for details. Abbreviations: Ac, acetyl; Ara*f*, arabinofuranose; Fuc, fucose; Gal, galactose; Glc, glucose; Xyl, xylose.

The XXXG-type xyloglucan in various angiosperm species bears acetyl groups predominantly at *O*-6 and, to a lesser extent, at *O*-3, *O*-4, *O*-3,4, and *O*-4,6 of side-chain Gal residues (Figure 2A) [15]. It was proposed that the *O*-acetyl groups at multiple carbon positions of the Gal residues could partially result from spontaneous migrations of *O*-acetyl groups [15]. Acetylation of the XXGG_n_-type xyloglucan in grasses and *Solanaceae* (e.g., tomato and tobacco) occurs at *O*-6 of unbranched backbone Glc residues (Figure 2B) [16,17]. The tomato XXGG_n_-type xyloglucan also has acetyl substitutions on side-chain Gal and Ara*f* residues at *O*-6 and *O*-5, respectively (Figure 2B) [16]. *O*-acetylation of unbranched backbone Glc residues has not been observed in the XXXG-type xyloglucan [13]. It is currently unknown whether xyloglucan in bryophytes, seedless vascular plants, and gymnosperms is acetylated, except that xyloglucan in the moss *Physcomitrium patens* was reported to lack acetyl groups [18,19].

*O*-acetylation of side-chain Gal residues in the XXXG-type xyloglucan is catalyzed by xyloglucan *O*-acetyltransferases named XGOATs, which are members of the TBL/DUF231 family (Figure 2C). In *Arabidopsis*, two XGOATs (AtXGOAT1/AXY4 and AtXGOAT2/AXY4L) are responsible for the *O*-acetylation of Gal residues of xyloglucan in different organs [20]. Mutation of the *AXY4* gene causes a loss of *O*-acetyl groups on xyloglucan in seedlings, leaves, and roots, whereas mutation of *AXY4L* results in a loss of *O*-acetyl groups on xyloglucan in seeds. In addition, a naturally occurring *Arabidopsis* ecotype, Ty-0, lacks *O*-acetyl groups on xyloglucan in rosette leaves and roots due to mutations in *AXY4* [20]. A rice AXY4 close homolog was shown to be able to acetylate xyloglucan when overexpressed in the *Arabidopsis axy4* mutant [21]. The biological functions of the *O*-acetylation of Gal residues of xyloglucan remain unclear as no visible phenotypes were observed in the *Arabidopsis axy4* and *axy4l* mutants.

Biochemical characterization of recombinant XGOATs produced in the human embryonic kidney (HEK) 293 cells has revealed their enzymatic properties. Recombinant *Arabidopsis* and poplar XGOATs, including AtXGOAT1/AXY4, AtXGOAT2/AXY4L, and PtrXGOAT1/2/3/4, catalyze predominantly 6-*O*-monoacetylation and, to a much lesser degree, 3-*O*- and 4-*O*- monoacetylation and 4,6-di-*O*-acetylation of Gal residues on xyloglucan (Figure 2D) [22], an *O*-acetylation pattern similar to that observed in sycamore xyloglucan [15]. Furthermore, it was found that XGOATs specifically acetylated fucosylated Gal residues but were unable to acetylate non-fucosylated Gal residues on xyloglucan [22], which could explain why a lack of xyloglucan Gal fucosylation in the *Arabidopsis* xyloglucan fucosyltransferase mutants (*atfut1*/*mur2*) leads to a loss of xyloglucan acetylation [23]. Since acetyl groups are present on both fucosylated and non-fucosylated Gal residues in *Arabidopsis* xyloglucan [20], it was proposed that the acetylated, non-fucosylated Gal residues most likely resulted from acetylated, fucosylated Gal residues after the removal of fucose by the apoplastic α-fucosidase AXY8 [22], whose mutation was shown to cause increased fucosylation of Gal residues on xyloglucan [24]. *O*-acetyltransferases responsible for acetyl substitutions of side-chain Ara*f* residues in tomato xyloglucan still have not been identified. Furthermore, no XGOAT close homologs are present in the moss *P. patens*, the seedless vascular plant *Selaginella moellendorffii*, or the gymnosperms pine and spruce (Figure 1) [25]. It will be interesting to find out in what lineages of plants TBLs first acquired the ability to acetylate side-chain Gal residues of xyloglucan.

The *O*-acetylation of backbone Glc residues in the XXGG_n_-type xyloglucan is mediated by xyloglucan backbone 6-*O*-acetyltransferases (XyBATs), another group of TBL members (Figure 2C). Overexpression of *Brachypodium distachyon* BdXyBAT1 in *Arabidopsis* leads to the *O*-acetylation of xyloglucan backbone Glc residues and a T-DNA insertion mutation of *BdXyBAT1* in *Brachypodium*, causes a reduction in xyloglucan *O*-acetylation [27]. Enzymatic activity studies of recombinant XyBATs of *Brachypodium*, rice, and tomato produced in HEK293 cells have revealed that they catalyze the transfer of acetyl groups onto *O*-6 of xyloglucan backbone Glc residues (Figure 2E) and are able to acetylate two or three consecutive Glc residues [26]. Two consecutive acetylated backbone Glc residues have been observed in *Brachypodium* xyloglucan [27]. Furthermore, based on the observations that XyBATs act poorly on xylosylated glucan and that xyloglucan xylosyltransferases (XXTs) can efficiently xylosylate acetylated glucan, it was suggested that during the synthesis of the XXGG_n_-type xyloglucan in grasses and tomato, the nascent glucan chains might be first acetylated by XyBATs and then xylosylated by XXTs [26]. Although *Arabidopsis* xyloglucan has been shown to be an XXXG type with no acetyl groups on backbone Glc residues, two *Arabidopsis* XyBAT homologs exhibit *O*-acetyltransferase activity catalyzing the transfer of acetyl groups onto *O*-6 of xyloglucan backbone Glc residues [26], indicating that some specialized cell types in *Arabidopsis* might have the XXGG_n_-type xyloglucan with acetylated backbone Glc residues.

Although the exact mechanisms determining the xylosylation patterns of the XXXG- and XXGG_n_-type xyloglucans remain unknown, acetylation of backbone Glc residues by XyBATs may play a role in the xylosylation pattern of the XXGG_n_-type xyloglucan. Because both acetylation and xylosylation of xyloglucan backbone Glc occur at *O*-6, 6-*O*-acetylation of a Glc residue prevents its 6-*O*-xylosylation, and vice versa. Experimental evidence that a change in backbone Glc acetylation could cause an alteration in the xyloglucan xylosylation pattern came from the findings that a mutation of *BdXyBAT1* in *Brachypodium* leads to the generation of XXXG units in the XXGG_n_-type xyloglucan and conversely, overexpression of XyBATs in *Arabidopsis* results in the production of acetylated XXGG_n_ units, including XXGG, XXGG, XXGGG, and XXGGG (G denotes acetylated Glc), in the XXXG-type xyloglucan [26,27]. Further investigation of how acetylation and xylosylation of xyloglucan backbone Glc residues are coordinated will provide insights into the biochemical mechanism underlying the synthesis of the XXGG_n_-type xyloglucan.

## 3. Xylan *O*-Acetyltransferases

**Figure 3:**
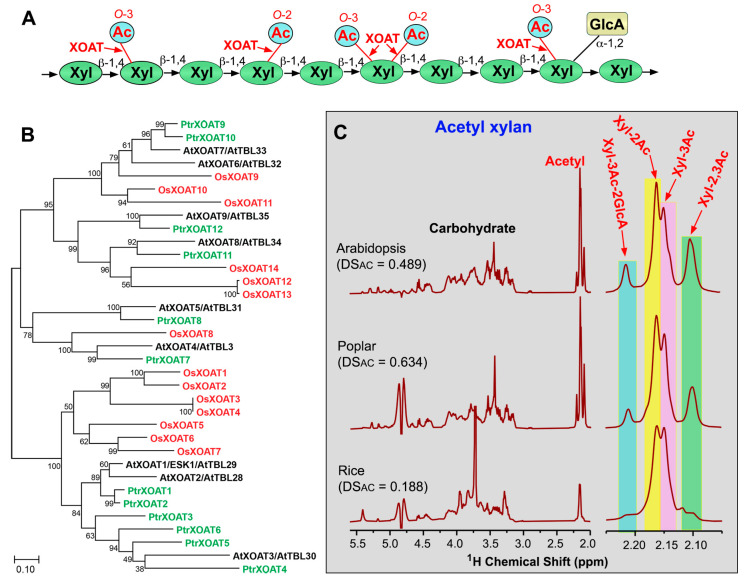
*O*-acetyltransferases mediating *O*-acetylation of xylan. (**A**) Diagram of the structural feature of xylan showing acetyl groups attached to *O*-2 and/or *O*-3 of Xyl residues. Abbreviations: Ac, acetyl; GlcA, glucuronic acid; Xyl, xylose. (**B**) Phylogenetic relationship of biochemically characterized XOATs from *Arabidopsis* (At), poplar (*Populus trichocarpa*; Ptr), and rice (*Oryza sativa*; Os). The phylogenetic tree was constructed using MEGA11 software with the maximum likelihood method. The numbers at the nodes represent bootstrap values as percentages of 1000 replicates and the 0.1 scale denotes 10% change. (**C**) ^1^H-NMR spectra of acetyl xylan isolated from *Arabidopsis*, poplar, and rice. Shown on the left are resonances corresponding to carbohydrate (3.0–5.5 ppm) and acetyl groups (2.0–2.25 ppm). Shown on the right are enlarged acetyl resonances attributed to acetyl groups attached to *O*-2 (Xyl-2Ac), *O*-3 (Xyl-3Ac), both *O*-2 and *O*-3 (Xyl-2,3Ac) of Xyl residues, and *O*-3 of 2-*O*-GlcA-substituted Xyl residues. See Zhong et al. (2017, 2018 and 2018) [35,36,47] for details. DS_AC_, degree of substitutions by acetyl groups.

Xylan is a major hemicellulosic polysaccharide present in the secondary walls of vascular plants. In grasses, it is also present in primary walls. Xylan in land plants consists of a backbone of β-1,4-linked D-Xyl residues that are often substituted at *O*-2 with α-D-glucuronic acid (GlcA) and/or 4-*O*-methyl-α-D-glucuronic acid (MeGlcA) side chains, depending on the species. In addition to GlcA/MeGlcA, xylan in gymnosperms is also substituted at *O*-3 with α-L-Ara*f* and that in grasses can also be substituted with various mono- and di-saccharide side chains, such as 2-*O*/3-*O*-α-L-Ara*f*, 2,3-di-*O*-α-L-Ara*f*, 2-*O*-β-D-Xyl-3-*O*-α-L-Ara*f*, and 2-*O*-α-L-Ara*f*-3-*O*-α-L-Ara*f* [28]. Xylan in the moss *P. patens*, the seedless vascular plant *S. moellendorffii* and various species of angiosperms have been shown to bear acetyl groups [29,30,31,32,33,34,35,36], whereas xylan in gymnosperms, except Gnetophytes, lacks acetyl groups [29,37]. The acetyl groups are attached to backbone Xyl residues; a Xyl residue may be mono-acetylated at *O*-2 (Xyl-2Ac) or *O*-3 (Xyl-3Ac), di-acetylated at both *O*-2 and *O*-3 (Xyl-2,3Ac), or acetylated at *O*-3 and GlcA/MeGlcA-substituted at *O*-2 (Xyl-3Ac-2GlcA) (Figure 3A) [30,33,34,38]. Although the ratio of acetylation at different carbon positions of Xyl varies between different plant species, xylan acetylation predominantly occurs at *O*-2 and *O*-3 of Xyl. For example, Xyl-2Ac, Xyl-3Ac, Xyl-2,3Ac, and Xyl-3Ac-2GlcA comprise 44%, 31%, 18%, and 7%, respectively, of the total acetylated Xyl in xylan isolated from *Arabidopsis*, 37%, 25%, 23%, and 14%, respectively, in that from poplar, and 45%, 40%, 9%, and 5%, respectively, in that from rice (Figure 3C) [35,36]. It is unclear whether these acetylation ratios represent the actual ones in native xylan as acetyl groups may migrate spontaneously between *O*-2 and *O*-3 positions of Xyl in isolated xylan in vitro [30]. The degree of substitutions by acetyl groups (DS_AC_) in xylan differs considerably among plant species. While the DS_AC_ of xylan in many dicot species, such as *Arabidopsis*, *Eucalyptus*, *Paulownia*, and poplar, is 50 to 60% [8,30,33,36,39], that of xylan in monocot species, such as rice, corn, and sugarcane, is much lower, ranging from 10 to 30% [35,40,41]. In *Arabidopsis* xylan, acetyl groups are mainly placed on every other Xyl residue, but domains with unevenly spaced acetyl groups also exist [8,42]. The even-spaced acetylation pattern is proposed to be critical for the proper interaction of xylan with the hydrophilic faces of cellulose microfibrils and hence is important for the normal assembly of secondary wall polymers [43].

Genetic and biochemical analyses have uncovered a group of TBL members as xylan *O*-acetyltransferases (XOATs) (Figure 3B). In *Arabidopsis*, nine AtXOATs are implicated in xylan *O*-acetylation. The mutation of *AtXOAT1* (also named *ESK1*/*TBL29*) causes a significant reduction in 2-*O*- and 3-*O*-mono-acetylation of xylan, mild collapse of xylem vessels, and an impairment in plant growth [6,8]. While a single mutation of other *AtXOATs* has no effects on xylan *O*-acetylation, double mutations of *AtXOAT4/5* (also named *TBL3/31*) result in a major reduction in xylan 3-*O*-monoacetylation [44], those of *AtXOAT6/7* (*TBL32/33*) reduce the levels of xylan 3-*O*-monoacetylation and 2,3-di-*O*-acetylation [7] and those of *AtXOAT8/9* (*TBL34/35*) lead to a complete loss of 3-*O*-acetylation of 2-*O*-GlcA/MeGlcA-substituted Xyl residues [45]. In addition, triple mutations of *AtXOAT1/6/7* (*ESK1/TBL32/TBL33*) or *AtXOAT1/8/9* (*ESK1/TBL34/TBL35*) cause a further drastic reduction in xylan acetylation levels, severe deformation of xylem vessels, an altered secondary wall structure, and stunted plant growth, demonstrating a crucial role of xylan *O*-acetylation in the normal secondary wall assembly and plant growth [7,45]. It is interesting to note that a reduction in xylan *O*-acetylation in the *xoat* mutants results in an increase in GlcA/MeGlcA substitutions of xylan [8] and conversely, simultaneous mutations of the glucuronoxylan glucuronyltransferases *GUX1* and *GUX2* lead to the increased acetylation of xylan [42,46]. Because GlcA/MeGlcA substitutions of xylan occur at the *O*-2 position of Xyl, it is conceivable that a reduction in xylan 2-*O*-acetylation would leave more *O*-2 of Xyl available for GlcA/MeGlcA substitutions and vice versa.

Enzymatic activity studies of recombinant XOATs expressed in HEK293 cells have revealed their regiospecificity in xylan *O*-acetylation. AtXOAT1/ESK1, AtXOAT2/TBL28, AtXOAT4/TBL3, AtXOAT5/TBL31, and AtXOAT8/TBL34 catalyze xylan 2-*O*- and 3-*O*-mono-acetylation and 2,3-di-*O*-acetylation; AtXOAT3/TBL30 carries out 2-*O*- and 3-*O*-mono-acetylation; AtXOAT9/TBL35 catalyzes 2,3-di-*O*-acetylation; and AtXOAT6/TBL32 and AtXOAT7/TBL33 specifically acetylate *O*-3 of 2-*O*-GlcA/MeGlcA-substituted Xyl residues [47,48]. Because acetyl groups may spontaneously migrate between *O*-2 and *O*-3 of Xyl [30], the relative levels of acetyl groups at *O*-2 and *O*-3 observed in the XOAT-catalyzed reaction products could be partially attributed to spontaneous acetyl migration. AtXOAT1 was shown to first add acetyl onto *O*-2 of Xyl and the acetyl groups could spontaneously migrate to *O*-3 of Xyl [48,49]. However, spontaneous acetyl migration is unlikely to occur in the case of 3-*O*-acetylation of 2-*O*-GlcA/MeGlcA-substituted Xyl catalyzed by AtXOAT6/7 because *O*-2 of Xyl is already occupied by GlcA/MeGlcA [47]. It remains unclear how 2,3-di-*O*-acetylation of a Xyl residue is carried out by XOATs, i.e., whether the Xyl residue is acetylated first at *O*-2 and then at *O*-3, or the first added acetyl at *O*-2 spontaneously migrates to *O*-3 and then another acetyl is added to *O*-2. Like AtXOATs, recombinant XOATs of rice and poplar have also been shown to catalyze 2-*O*- and 3-*O*-mono-acetylation and 2,3-di-*O*-acetylation of xylan, albeit with differential positional preferences [35,36]. Although xylan in *P. patens* and *S. moellendorffii* also bear acetyl groups [31], none of their TBL members are phylogenetically grouped together with XOATs (Figure 1) [28]. It will be interesting to find out whether other TBL members are responsible for xylan *O*-acetylation in *P. patens* and *S. moellendorffii*.

## 4. Mannan *O*-Acetyltransferases

**Figure 4:**
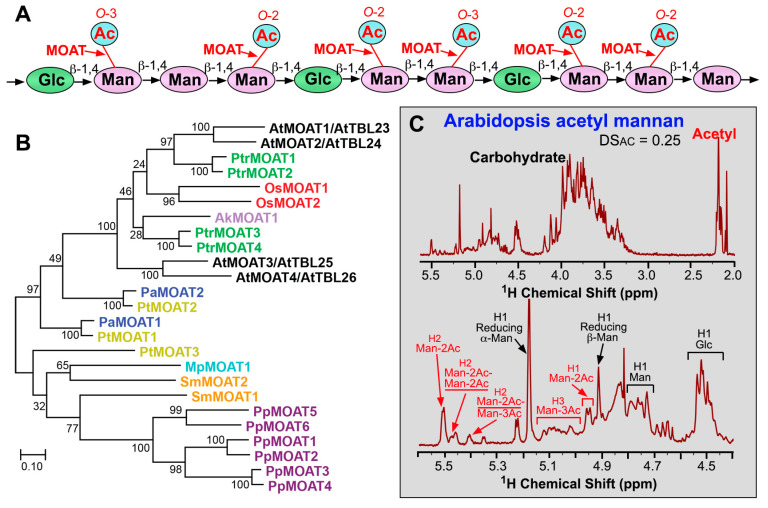
*O*-acetyltransferases mediating *O*-acetylation of mannan. (**A**) Diagram of the structural feature of glucomannan showing acetyl groups attached to *O*-2 or *O*-3 of Man residues. Abbreviations: Ac, acetyl; Glc, glucose; Man, mannose. (**B**) Phylogenetic relationship of biochemically characterized MOATs from *Arabidopsis* (At), poplar (*Populus trichocarpa*; Ptr), rice (*Oryza sativa*; Os), voodoo lily (*Amorphophallus Konjac*; Ak), pine (*Pinus taeda*; Pt), spruce (*Picea abies*; Pa), *Selaginella moellendorffii* (Sm), moss (*Physcomitrium Patens*; Pp), and *Marchantia polymorpha* (Mp). The phylogenetic tree was constructed using MEGA11 software with the maximum likelihood method. The numbers at the nodes represent bootstrap values as percentages of 1000 replicates and the 0.1 scale denotes 10% change. (**C**) ^1^H-NMR spectra of acetyl mannan isolated from *Arabidopsis*. The top panel shows resonances corresponding to carbohydrate (3.0–5.5 ppm) and acetyl groups (2.0–2.25 ppm). The bottom panel displays enlarged resonances attributed to Man residues acetylated at *O*-2 (Man-2Ac) or *O*-3 (Man-3Ac). Man-2Ac-Man-2Ac and Man-2Ac-Man-3Ac refer to resonances corresponding to the 2-O-acetylated Man (underlined) in two consecutive acetylated Man residues. See Zhong et al. (2018) [61] for details. DS_AC_, degree of substitutions by acetyl groups.

Mannan is a hemicellulosic polysaccharide found in both primary and secondary walls of plants and it is abundant in the cell walls of bryophytes, seedless vascular plants, and gymnosperms [37,50,51,52,53]. Based on its chemical structure, mannan is classified into homomannan, glucomannan, galactomannan, and galactoglucomannan. Homomannan and galactomannan are composed of a linear chain of β-1,4-linked D-mannosyl (Man) residues that may be unsubstituted (homomannan) or substituted at *O*-6 with α-D-Gal (galactomannan). Glucomannan and galactoglucomannan consist of a linear chain of β-1,4-linked D-Man residues interspersed with β-1,4-linked D-Glc residues, which may be unsubstituted (glucomannan) or substituted at *O*-6 of Man with α-D-Gal (galactoglucomannan) [54,55]. Mannan from bryophytes, seedless vascular plants, gymnosperms, and angiosperms has been found to be acetylated predominantly at *O*-2 and *O*-3 of Man residues (Figure 4A,C). The acetyl groups can be attached to two adjacent Man residues. Low levels of 6-*O*-, 2,3-di-*O*-, 2,6-di-*O*-, and 3,6-di-*O*-acetyl substitutions were also present in the glucomannan of *Dendrobium officinale* [56]. The degree of *O*-acetylation in mannan from different lineages of plant species, including *P. patens*, *S. moellendorffii*, pine, spruce, *Arabidopsis*, flax, birch, and aspen, ranges from 0.22 to 0.37 (Figure 4C) [25,57,58,59,60,61].

Mannan *O*-acetylation has been shown to be catalyzed by mannan *O*-acetyltransferases (MOATs), another group of TBL members (Figure 4B). Deep sequencing of *Amorphophallus konjac* corm, which is rich in glucomannan, identified a close homolog of *Arabidopsis* TBL25 among the top 10 most abundantly expressed transcripts, suggesting its possible association with mannan synthesis [20]. An investigation of enzymatic activities of recombinant TBL proteins expressed in HEK293 cells has demonstrated that four *Arabidopsis* TBLs, including AtMOAT1/TBL23, AtMOAT2/TBL24, AtMOAT3/TBL25 and AtMOAT4/TBL26, and an *A. konjac* TBL25 homolog, AkMOAT1, are mannan *O*-acetyltransferases catalyzing 2-*O*- and 3-*O*-acetylation of mannan [61]. Simultaneous RNAi interference (RNAi) downregulation of *AtMOAT1/2/3/4* expression in *Arabidopsis* causes a drastic reduction in the degree of acetyl substitutions in mannan, providing genetic evidence for their roles in mannan *O*-acetylation [61]. However, no apparent morphological and cell wall phenotypes were observed in these transgenic *Arabidopsis* plants deficient in mannan *O*-acetylation. Recombinant proteins of MOAT close homologs from different lineages of plants, including *Marchantia polymorpha* (a liverwort), *P. patens*, *S. moellendorffii*, pine, spruce, rice, and poplar, also exhibit *O*-acetyltransferase activities catalyzing the transfer of acetyl groups to *O*-2 and *O*-3 of mannan, indicating that the biochemical functions of MOATs are evolutionarily conserved throughout land plants (Figure 4B) [25,62].

## 5. Pectin *O*-Acetyltransferases

**Figure 5:**
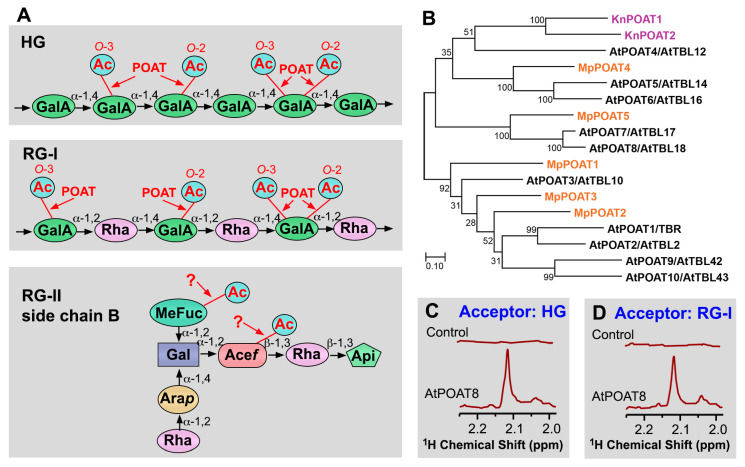
*O*-acetyltransferases mediating *O*-acetylation of pectins. (**A**) Diagrams of the structural units of the pectins HG and RG-I and the side chain B of RG-II showing acetyl groups attached to *O*-2 and/or *O*-3 of GalA residues in HG and RG-I and to the side-chain MeFuc and Ace*f* residues in RG-II. The question marks indicate that the corresponding *O*-acetyltransferases have not yet been identified. Abbreviations: Ac, acetyl; Acef, aceric acid; Api, apiose; Ara*p*, arabinopyranose; Gal, galactose; GalA, galacturonic acid; MeFuc, 2-*O*-methylfucose; Rha, rhamnose. (**B**) Phylogenetic relationship of biochemically characterized POATs from *Arabidopsis* (At), *Marchantia polymorpha* (Mp), and *Klebsormidium nitens* (Kn). The phylogenetic tree was constructed using MEGA11 software with the maximum likelihood method. The numbers at the nodes represent bootstrap values as percentages of 1000 replicates and the 0.1 scale denotes 10% change. (**C**) ^1^H-NMR spectra of the acetyl resonance region of unacetylated HG (control) and acetylated HG catalyzed by AtPOAT8 showing resonances attributed to acetyl groups. (**D**) ^1^H-NMR spectra of the acetyl resonance region of unacetylated RG-I (control) and acetylated RG-I catalyzed by AtPOAT8 showing resonances attributed to acetyl groups. See Zhong et al. (2024) [73] for details.

Pectins are a group of GalA-containing polysaccharides ubiquitously present in the primary wall and middle lamella of plant cells and they include homogalacturonan (HG), rhamnogalacturonan-I (RG-I), and rhamnogalacturonan-II (RG-II). HG and RG-II consist of a linear chain of α-1,4-linked GalA residues that may be unsubstituted (HG) or substituted with complex side chains (RG-II), and RG-I is made up of repeating units of [→α-GalA-1,2-α-Rha-1,4→] in which rhamnose (Rha) is often substituted with various side chains [63,64]. Structural analyses of pectins isolated from a number of dicot plants, including potato, tobacco, tomato, carrot, cotton, sugar beet, spinach, sycamore, and okra, have revealed that the *O*-acetylation of HG and RG-I occurs at *O*-2 and/or *O*-3 of backbone GalA residues (Figure 5A) [65,66,67,68,69,70,71]. In okra RG-I, acetyl substituents were also detected at *O*-3 of backbone Rha residues [71]. On the other hand, *O*-acetylation of RG-II occurs on 2-*O*-methyl-Fuc and aceric acid residues of its side chains rather than backbone GalA residues (Figure 5A) [72]. Currently, little is known about the status of the *O*-acetylation of pectins in other lineages of plants except dicots.

Mutational studies have implicated three *Arabidopsis* TBL genes, including *TBR*, *TBL10*, and *PMR5* (*powdery mildew resistant 5*)/*TBL44*, in pectin *O*-acetylation. The *tbr* mutant was identified during the screening of *Arabidopsis* mutants defective in trichome birefringence due to reduced crystalline cellulose [74]. The *tbr* mutation was found to occur in a member of the DUF231 family, and thus the DUF231 family was named the TBR-Like (TBL) family with TBR as the founding member [75]. In addition to reduced crystalline cellulose, the *tbr* mutant also has reduced pectin acetylation but elevated pectin methylesterification, and hence TBR was proposed to function in transferring acetyl groups onto pectins or protecting *O*-acetylated pectins from de-acetylation by pectin acetylesterases [75,76]. The *TBL10* mutation causes a significant reduction in RG-I *O*-acetylation, leading to the suggestion that it is an *O*-acetyltransferase acetylating RG-I or it provides an acetylated intermediate for RG-I *O*-acetylation [77]. The *pmr5* mutant confers enhanced resistance to powdery mildew and carries a mutation in the *TBL44* gene [11]. The *pmr5* mutant had a reduced cell wall acetyl content and the recombinant PMR5 protein expressed in *Escherichia coli* was shown to transfer acetyl groups from acetyl-CoA onto oligogalacturonides [9].

Comprehensive biochemical characterization of recombinant *Arabidopsis* TBL proteins expressed in HEK293 cells has uncovered ten TBL members as pectin *O*-acetyltransferases (POATs) (Figure 5B). They exhibit differential activities toward HG and RG-I: AtPOAT2/TBL2, AtPOAT4/TBL12, AtPOAT9/TBL42, and AtPOAT10/TBL43 transfer acetyl groups onto HG; AtPOAT5/TBL14 and AtPOAT6/TBL16 acetylate RG-I; and AtPOAT1/TBR, AtPOAT3/TBL10, AtPOAT7/TBL17, and AtPOAT8/TBL18 are able to act on both HG and RG-I (Figure 5C,D) [73]. Simultaneous RNAi downregulation of the expression of AtPOAT1/3/6/7/8, which show relatively high *O*-acetyltransferase activities toward HG and/or RG-I, leads to reduced pectin *O*-acetylation and altered plant growth in *Arabidopsis* [73]. The biochemical proof of AtPOAT1/TBR and AtPOAT3/TBL10 as pectin *O*-acetyltransferases is congruent with the genetic evidence that their mutations result in reduced pectin *O*-acetylation [75,76,77]. The finding that a suite of *Arabidopsis* TBLs are pectin *O*-acetyltransferases indicates that like that of other wall polysaccharides, the *O*-acetylation of pectins also entails multiple, functionally redundant *O*-acetyltransferases. Enzymatic activity studies of recombinant TBL proteins of *Klebsormidium nitens* (a charophyte green alga) and *M. polymorpha* have revealed that the two *K. nitens* TBL proteins (KnPOAT1/2) are *O*-acetyltransferases acetylating HG and five *M. polymorpha* TBLs (MpPOAT1 to 5) possess acetyltransferase activities toward HG and/or RG-I (Figure 5B) [62]. *O*-acetyltransferases responsible for acetylation of the side chain 2-*O*-methyl-Fuc and aceric acid residues of RG-II have yet to be identified. Considering that all known cell wall polysaccharide *O*-acetyltransferases belong to the TBL family, it is tempting to propose that some TBL members are involved in RG-II *O*-acetylation.

## 6. Structure and Mechanism of Action of Plant Cell Wall Polysaccharide *O*-Acetyltransferases

Sequence analysis of the 46 members of the *Arabidopsis* TBL family revealed the presence of two conserved domains, TBL and DUF231, which harbor the conserved GDS (Gly-Asp-Ser) and DXXH (Asp-X-X-His) motifs, respectively [75]. The predicted structure model of TBR shows that Ser in the GDS motif and Asp and His in the DXXH motif structurally align with the known Ser-His-Asp catalytic triad of the fungal *Aspergillus aculeatus* rhamnogalacturonan acetylesterase [78], a member of the SGNH family in the GDSL superfamily of esterases/lipases [79,80]. Structural and mutational analyses of the bacterial peptidoglycan *O*-acetyltransferases NgPatB and SaOatA_c_ have demonstrated that they possess a Ser-His-Asp catalytic triad, and mutations of these conserved amino acid residues impair their *O*-acetyltransferase activities [81,82]. It has been proposed that the mechanism of action of bacterial glycan *O*-acetyltransferases involves the Ser-His-Asp catalytic triad and employs a double-displacement, ping-pong bi-bi reaction, analogous to that of the well-characterized serine esterases [82,83]. In this two-step process, *O*-acetyltransferases first bind and transfer the acetyl group to the catalytic Ser residue to form a covalent acetyl–enzyme intermediate. Upon the binding of the glycan acceptor to the acetylated enzyme, the acetyl group is then transferred to the hydroxyl group of the glycan acceptor [84]. Covalently linked acetyl group to the catalytic Ser residue has been observed in several bacterial peptidoglycan *O*-acetyltransferases, including NgPatB, SpOatA, and SaOatA_c_ [81,82,83].

**Figure 6:**
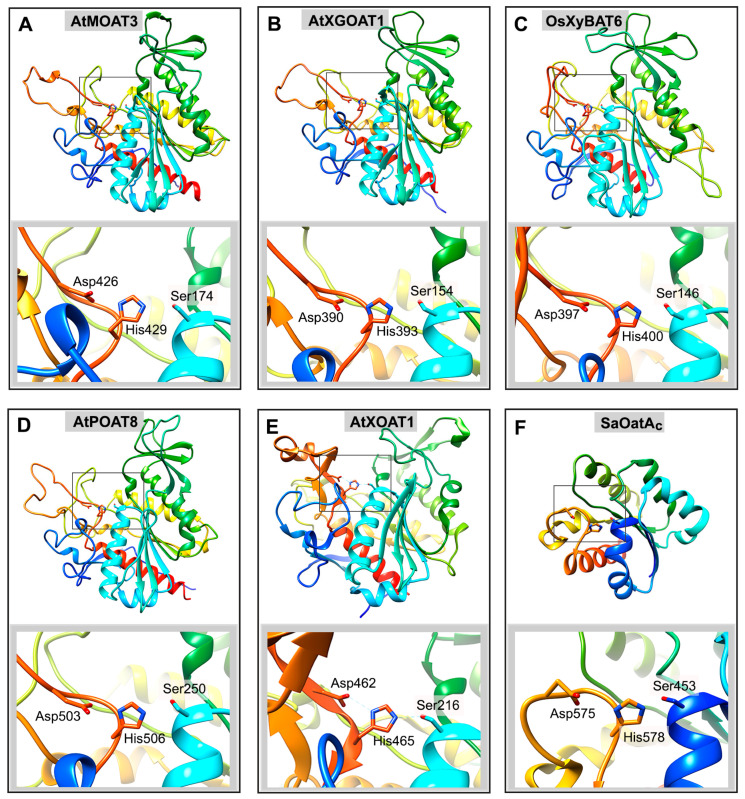
Structural similarities of plant cell wall polysaccharide *O*-acetyltransferases. The structure of the bacterial peptidoglycan *O*-acetyltransferase SaOatA_c_ is shown for comparison. The structural models of the catalytic domains of AtMOAT3 (amino acids 92-456), AtXGOAT1 (amino acids 72-416), OsXyBAT6 (amino acids 61-420), and AtPOAT8 (amino acids 168-533) were predicted using AlphaFold2, and the structures of the catalytic domains of AtXOAT1 (amino acids 133-487) and SaOatA_c_ (amino acids 445-601) were obtained from the Protein Data Bank (6CCI and 6VJP, respectively). The top panels display the whole view of the structural models of AtMOAT3 (**A**), AtXGOAT1 (**B**), OsXyBAT6 (**C**), AtPOAT8 (**D**), AtXOAT1 (**E**), and SaOatA_c_ (**F**). The bottom panels show close-up views of the active site of each protein from the boxed area in the top panel. The conserved Ser-His-Asp catalytic triad at the active site of each protein is highlighted.

Biochemical studies of plant cell wall polysaccharide *O*-acetyltransferases demonstrated that like bacterial glycan *O*-acetyltransferases, they also evolved to employ the Ser-His-Asp catalytic triad for their mechanism of action. Mutations of the conserved amino acid residues in the GDS and DXXH motifs of AtXOAT1/ESK1, AtMOAT3, AtXGOAT1/AXY4, AtPOAT3, and AtPOAT8 all result in a loss of their ability to transfer acetyl groups onto their respective acceptors, supporting the critical role of the Ser-His-Asp catalytic triad in their *O*-acetyltransferase activities [22,47,49,61,73]. Structural analysis of AtXOAT1 revealed its structural similarity with bacterial peptidoglycan *O*-acetyltransferases and the placement of the Ser-His-Asp catalytic triad in the predicted active site [49]. The structural models of representatives of other plant cell wall polysaccharide *O*-acetyltransferases, including AtMOAT3, AtXGOAT1, OsXyBAT6, and AtPOAT8, share with AtXOAT1 similar overall structural folds and positioning of the Ser-His-Asp catalytic triad in their predicted active sites (Figure 6). Like bacterial glycan *O*-acetyltransferases, the catalytic Ser residue of AtXOAT1 was also shown to be acetylated upon incubation with the acetyl donor to form a covalent acetyl–enzyme intermediate [49], indicating conservation of the double-displacement, ping-pong bi-bi reaction mechanism for plant cell wall polysaccharide *O*-acetylation.

## 7. Roles of RWAs and AXY9 in Plant Cell Wall Polysaccharide *O*-Acetylation

In addition to *O*-acetyltransferases, two additional groups of proteins, including RWAs and AXY9, are involved in plant cell wall polysaccharide *O*-acetylation. RWAs are homologs of CAS1 (capsule synthesis 1) of the fungal *Cryptococcus neoformans*, which is essential for *O*-acetylation of the capsular polysaccharide glucuronoxylomannan [85]. Mutation of the *CAS1* gene causes a loss of acetyl groups in glucuronoxylomannan, and it was proposed that the CAS1 protein is a membrane-associated glucuronoxylomannan *O*-acetyltransferase [85]. The four *Arabidopsis RWA* genes are highly expressed in secondary wall-forming cells, and their simultaneous T-DNA knockout mutations result in a 40% reduction in xylan *O*-acetylation as well as reduced *O*-acetylation in other cell wall polysaccharides, including xyloglucan, mannan, and pectins [32,86,87], indicating that RWAs play a role common for the *O*-acetylation of all plant cell wall polysaccharides. Likewise, RNAi downregulation of RWA genes in transgenic poplar plants causes the reduced *O*-acetylation of wood xylan and xyloglucan [88]. While the fungal CAS1 protein consists of a domain with multiple (>10) transmembrane helices and a large globular putative *O*-acetyltransferase domain [85], plant RWA proteins only contain multiple transmembrane helices without a putative *O*-acetyltransferase domain, and hence they are unlikely to directly catalyze the acetylation of plant cell wall polysaccharides.

An incubation of potato microsomes with radiolabeled acetyl-CoA was shown to result in the generation of radiolabeled cell wall polysaccharides, including xyloglucan, HG, and RG-I, implying that acetyl-CoA is an acetyl donor for the *O*-acetylation of plant cell wall polysaccharides [89]. Acetyl-CoA in plant cells is synthesized in four different subcellular compartments, including cytosol, mitochondrion, plastid, and peroxisome [90], and cytosolic acetyl-CoA generated by ATP-citrate lyases is crucial for the *O*-acetylation of cell wall polysaccharides, including xylan, xyloglucan, mannan, and pectins [91]. Since the Golgi is not known to contain an acetyl-CoA-generating pathway and lipid membranes are impermeable to acetyl-CoA [90], it is conceivable that cytosolic acetyl-CoA must be translocated by transporters into the Golgi, where cell wall polysaccharide *O*-acetylation occurs. Simultaneous mutations of the four *Arabidopsis RWAs* were found to cause a drastic reduction in acetyl-CoA transport across the Golgi membranes [91], supporting the hypothesis that RWAs might be involved in translocating acetyl-CoA from the cytosol into the Golgi [20]. Direct proof of RWAs as acetyl-CoA transporters requires further evidence.

Similar to RWAs, the *Arabidopsis* AXY9 protein is also essential for the *O*-acetylation of multiple cell wall polysaccharides because its mutation reduces the acetylation of xyloglucan, xylan, and pectins. AXY9 shares a low sequence similarity with TBLs, and it also contains the conserved GDS and DXXH motifs [92]. An activity assay of the recombinant AXY9 protein expressed in HEK293 cells showed that it was unable to transfer acetyl groups from acetyl-CoA onto xyloglucan, xylan, or mannan, indicating that it does not directly catalyze acetyl transfer onto cell wall polysaccharides. However, AXY9 possesses weak acetylesterase activity toward esterase pseudosubstrates, suggesting that it is catalytically active [22]. The weak acetylesterase activity of AXY9 concurs with the proposition that AXY9 might act as an intermediate for the transfer of acetyl groups from an acetyl donor to cell wall polysaccharide *O*-acetyltransferases [92]. The exact function of AXY9 in plant cell wall polysaccharide *O*-acetylation awaits further elucidation of its biochemical activity.

The involvement of multiple groups of proteins, including TBLs, RWAs, and AXY9, in the plant cell wall polysaccharide *O*-acetylation resembles the *O*-acetylation machinery for the bacterial alginate, a linear polysaccharide composed of β-1,4-linked D-mannuronic acid and α-L-guluronic acid. Four proteins, including AlgI, AlgJ, AlgF, and AlgX, are implicated in bacterial alginate *O*-acetylation [93]. AlgI, which is located in the inner membrane, is proposed to transport the acetyl donor from the cytosol through the inner membrane into the periplasm, where alginate *O*-acetylation occurs. AlgJ, an SGNH hydrolase-like protein containing the conserved catalytic Asp-His-Ser triad, which exhibits acetylesterase activity but no *O*-acetyltransferase activity, is proposed to function as an intermediate to shuttle the acetyl group from AlgI to AlgX [93]. AlgX, also an SGNH hydrolase-like protein with the conserved catalytic Asp-His-Ser triad, is an *O*-acetyltransferase mediating acetyl transfer onto alginate [94]. Although AlgF is also required for alginate *O*-acetylation, its actual function is currently unknown [93]. The *O*-acetylation machinery of plant cell wall polysaccharides is somewhat analogous to that of the bacterial alginate; RWAs may function similarly to AlgI in translocating the acetyl donor across the membrane, AXY9 may be similar to AlgJ acting as an intermediate in shuttling the acetyl group to *O*-acetyltransferases, and TBLs are equivalent to AlgX in catalyzing the acetylation of polysaccharide acceptors. The resemblance of polysaccharide *O*-acetylation machineries between plants and acetylated alginate-producing bacteria may be resulted from convergent evolution.

## 8. Evolutionary Origins of TBLs, RWAs and AXY9

TBL members are present throughout different lineages of land plants, ranging from nonvascular and seedless vascular plants to seed plants (gymnosperms and angiosperms). The TBL family underwent a large expansion during the divergence of seed plants, resulting in an emergence of several new clades (Figure 1) [25]. One of them is the XOAT clade catalyzing xylan *O*-acetylation, and it appeared to be specific to angiosperms. TBL homologs also exist in the genomes of a number of charophyte green algae [25], which are the closest algal relatives of land plants [95], indicating that TBLs in land plants may share a common ancestor with those in charophyte green algae.

Biochemical characterization of recombinant proteins of TBLs from *K. nitens*, a representative of an early divergent class of charophyte green algae [96], and *M. polymorpha*, a liverwort that is an extant representative of an ancient lineage of land plants [97], has demonstrated that the two *K. nitens* TBLs and five of the six *M. polymorpha* ones exhibit *O*-acetyltransferase activities toward pectins, and one *M. polymorpha* TBL is a mannan *O*-acetyltransferase [62]. These findings suggest that ancestral TBLs were first recruited as polysaccharide *O*-acetyltransferases as early as in charophyte green algae to acetylate pectins, and when ancient lineages of land plants emerged, TBL genes were expanded via gene duplication and functional diversification to acetylate additional wall polysaccharides, first mannan and then xylan and xyloglucan, during the evolution of land plants [62]. Homologs of RWAs and AXY9 are also present in different lineages of land plants as well as in charophyte green algae [62]. It has yet to be investigated whether these homologs in charophyte green algae were also recruited as players in cell wall polysaccharide *O*-acetylation.

## 9. Plant Cell Wall Polysaccharide *O*-Acetylesterases

Little is known about whether any plant acetylesterases are involved in modulating the degree of *O*-acetylation of cell wall polysaccharides, except pectins, after they are secreted into the extracellular space. Plant pectin acetylesterases (PAEs) are localized in apoplasts and they hydrolyze acetyl groups from acetylated pectins [98,99]. They are widely distributed among different lineages of land plants, ranging from bryophytes (*P. patens*) and seedless vascular plants (*S. moellendorffii*) to gymnosperms (pine) and angiosperms (monocots and dicots) [100,101,102]. PAEs are members of the carbohydrate esterase family 13 (CE13), which differs from the bacterial and fungal pectin acetylesterases residing in the CE12 family, although both CE12 and CE13 belong to the SGNH hydrolase family. Like typical SGNH family hydrolases, PAEs across all plant kingdoms possess a putative Ser-His-Asp catalytic triad for their hydrolytic activity [102].

Multiple PAE genes exist in the genomes of different plant species [102]. It is currently unclear whether different PAEs exhibit differential substrate specificities toward HG, RG-I, and RG-II. The *Arabidopsis* genome harbors 12 PAEs, and mutations of two of them, *PAE8* and *PAE9*, were shown to cause an increase in acetate content in pectins, consistent with their proposed roles in modulating the degree of pectin *O*-acetylation [103]. More elevated acetate content was observed in the pectic fractions enriched with RG-I than those enriched with HG in the *pae8* and *pae9* mutants [103], suggesting that PAE8 and PAE9 may preferentially act on acetylated RG-I, although their substrate specificity awaits further biochemical study.

In addition to PAEs, an *Arabidopsis* TBL member, TBL38, was suggested to be a cell wall-localized pectin acetylesterase [104], a biochemical property different from the many TBLs that are Golgi-localized cell wall polysaccharide *O*-acetyltransferases. A mutation of the *TBL38* gene was shown to cause an increased acetate content in HG but not RG-I in seedcoat cell walls and its recombinant protein expressed in *Pichia pastoris* was able to release acetate from acetylated pectins, leading to the conclusion that TBL38 is an HG acetylesterase [104]. It will be interesting to find out whether any other TBLs with unknown functions may also function as cell wall-localized acetylesterases acting on acetylated RG-I, RG-II or hemicelluloses.

Although cell wall-localized acetylesterases acting on acetylated hemicelluloses, including xylan, mannan, and xyloglucan, have not been identified in plants, two rice Golgi-localized proteins, OsBS1 (brittle leaf sheath1) and OsDARX1 (deacetylase on arabinosyl sidechain of xylan1), belonging to the GDSL-lipase/esterase family, were implicated in xylan deacetylation [105,106]. Mutation of the *OsBS1* gene resulted in fragile leaf sheath, reduced plant growth, and increased xylan backbone acetylation. Recombinant OsBS1 protein expressed in *Pichia* was shown to hydrolyze acetyl groups from acetylated xylan. It was concluded that OsBS1 was an acetylesterase cleaving acetyl groups attached to the xylan backbone [105]. On the other hand, OsDARX1 was suggested to be an acetylesterase acting on acetyl groups attached to Ara*f* side chains of xylan. A mutation of the *OsDARX1* gene led to an increase in the acetylation of xylan Ara*f* side chains and the recombinant OsDARX1 protein expressed in *Pichia* released acetate from xylooligomers isolated from the *darx1* mutant [106]. Since both OsBS1 and OsDARX1 are localized in the Golgi [105,106], it appears that they modulate xylan acetylation at the same time when xylan is acetylated by XOATs during xylan synthesis, which differs from the cell wall-localized PAEs. There are a number of close homologs of OsBS1 and OsDARX1 in rice and it will be interesting to examine whether they are also acetylesterases involved in modulating cell wall polysaccharide acetylation. In addition, it is not known whether modulation of xylan *O*-acetylation in the Golgi is specific to grass species or a common mechanism in plants.

## 10. Biological Functions of Plant Cell Wall Polysaccharide *O*-Acetylation

The *O*-acetylation of cell wall polysaccharides, such as xylan and pectins, has been shown to play important roles in plant growth, biotic and abiotic stress responses, and cell wall physico-chemical properties. *Arabidopsis* mutants deficient in xylan *O*-acetylation, such as the *esk1* mutant, the *esk1 tbl32 tbl33*, and the *esk1 tbl34 tbl35* triple mutants, exhibited various phenotypes, including stunted plant growth, increased freezing, salt, and drought tolerance [107,108,109], collapsed xylem vessels [6,8,110], and reduced cellulose and xylan deposition and altered secondary wall structure [7,45]. The stunted plant growth and collapsed xylem vessel phenotypes in these mutants are reminiscent of the *Arabidopsis* mutants defective in the synthesis of the xylan backbone or its unique tetrasaccharide reducing end sequence [28], indicating that an alteration in the xylan amount or acetylation causes common phenotypes. Interestingly, the developmental phenotypes of the *esk1* mutant are suppressed by mutation of the *ubiquitin protein ligase3* gene [111] or the *MAX4* strigolactone biosynthetic gene [112]. The finding that blocking strigolactone synthesis suppresses the *esk1* developmental phenotypes indicates the involvement of the strigolactone hormonal pathway in response to xylan *O*-acetylation deficiency [112].

It has been shown that in *Arabidopsis*, the acetyl moieties are evenly distributed along the xylan backbone and xylan adopts a flattened, ribbon-like, twofold screw conformation when bound to cellulose microfibrils in the cell wall [43]. A disruption of the even-patterned acetyl substitutions of xylan was observed in the *esk1* mutant, which rendered the mutant xylan unable to interact normally with cellulose microfibrils. It was proposed that the even-patterned acetyl substitutions together with the GlcA side chains enable xylan to interact with the hydrophilic surfaces of cellulose microfibrils, hence allowing normal secondary wall assembly [43]. The aberrant secondary wall structure caused by a deficiency in xylan *O*-acetylation most likely weakens cell wall strength, thus leading to collapsed xylem vessels and the subsequent impairment of plant growth and elicitation of stress responses. In addition, it was found that xylan isolated from the *Arabidopsis gux1/2* mutant deficient in GlcA side chains by alkali extraction, which removes acetyl groups, was insoluble, whereas DMSO-extracted *gux1/2* xylan, that retains acetyl groups, remained soluble [46,113], indicating that acetyl groups are critical for xylan solubility.

*Arabidopsis* mutants defective in pectin *O*-acetylation also displayed various developmental and stress response phenotypes. For example, the *tbr* mutant has reduced crystalline cellulose, increased pectin methylesterification, and altered photomorphogenesis [75,76]; the *tbl10* mutant shows enhanced drought tolerance [77], the *pmr5* mutant exhibits resistance to powdery mildew and a reduction in leaf cell expansion [9,11], and the simultaneous RNAi downregulation of At*POAT1/3/6/7/8* in *Arabidopsis* causes an impairment of plant growth, including reduced cell expansion, decreased flower filament elongation, and production of infertile siliques [73]. Moreover, a reduction in pectin *O*-acetylation in tobacco by overexpression of poplar PAE1 impairs cell elongation, pollen germination, and plant reproduction [10], and overexpression of a mung bean PAE in potato tubers results in a stiffer tuber tissue and a stronger cell wall matrix [114]. Because pectin crosslinking by calcium is important for modulating cell wall mechanics and acetyl groups in pectins hinder dimerization of pectic chains by calcium [115,116,117,118], the impaired cell elongation in plants with reduced pectin *O*-acetylation could be attributed to an increase in pectin crosslinking, and hence cell wall stiffening. It is intriguing that a decrease in pectin *O*-acetylation could confer resistance to fungal pathogens [9,11], which may be due to the constitutive activation of defense mechanisms as seen in other cell wall mutants [119,120,121]. Increased resistance to fungal pathogens was also observed in the *Arabidopsis rwa2* mutant and overexpressors of a fungal PAE, which had reduced *O*-acetylation of both pectins and hemicelluloses [86,122].

In contrast to xylan and pectins, a reduction in *O*-acetylation of xyloglucan or mannan has no apparent adverse impacts on plant growth. The *Arabidopsis axy4* and *axy4l* mutants, which are deficient in *O*-acetylation of Gal side chains of xyloglucan, exhibit no visible phenotypes compared with the wild type [20]. Likewise, *Arabidopsis* plants with simultaneous RNAi downregulation of MOAT1/2/3/4, which have an 84% reduction in mannan *O*-acetylation, show no obvious defects in plant growth and development [61]. Xyloglucan has been shown to be critical for aluminum binding in *Arabidopsis* root cell walls and the level of bound aluminum is elevated in root cell walls of the *axy4* mutant, suggesting that *O*-acetylation of xyloglucan affects its aluminum binding capacity [123]. Mannan exhibits high binding affinity to cellulose [124] and an in vitro sorption study revealed that mannan deacetylation dramatically increased its sorption onto bleached kraft pulp [125], indicating that the *O*-acetylation of mannan may influence its affinity for cellulose. Since acetylated mannan is a major component in gymnosperm wood [25,57,59,60], it will be interesting to investigate whether mutations of MOATs in gymnosperms, such as pine and spruce, would have any impacts on secondary wall assembly.

## 11. Biotechnological Applications of Manipulations of Plant Cell Wall Polysaccharide *O*-Acetylation

Plant cell walls, often coined lignocellulosic biomass, have been exploited as a renewable source of second-generation biofuel production. One of the major obstacles for economic biofuel production is biomass recalcitrance, i.e., the resistance of digestion of cell walls into fermentable sugars by cell wall-degrading enzymes, and one factor contributing to biomass recalcitrance is the acetylation of cell wall polysaccharides [126]. It has been shown that cell wall acetylation adversely affects the hydrolysis of cell walls into fermentable sugars by cellulolytic and xylanolytic enzymes [127], and acetate released from the pretreatment of biomass is inhibitory to microorganisms used for the fermentation of sugars [128]. It is envisaged that a reduction in cell wall acetylation would be beneficial to biomass conversion into biofuels; a 20% reduction in cell wall acetylation is estimated to result in a 10% decrease in the production costs of ethanol [129]. Consistent with this proposition, it was shown that a reduction in cell wall *O*-acetylation in *Arabidopsis* by overexpression of fungal acetylxylan esterases produced more fermentable sugars by the enzymatic hydrolysis of cell walls and yielded more ethanol by the fungal fermentation of acid hydrolysates of cell walls [130,131]. Similarly, a reduction in cell wall *O*-acetylation in transgenic poplar by RNAi downregulation of RWA genes or overexpression of fungal acetylxylan esterases was found to improve the enzymatic release of glucose from transgenic wood [88,132,133]. Transgenic plants with reduced cell wall *O*-acetylation also have an increased resistance to fungal pathogens [11,86,122,131,134]), which may provide additional benefits for biofuel production. By contrast, an increase in cell wall *O*-acetylation in transgenic poplar by overexpression of a poplar RWA gene caused a reduction in the release of fermentable sugars by the enzymatic hydrolysis of cell walls [135]. Since chemically acetylated wood has enhanced mechanical strength, durability, and resistance to fungi and termites [4], genetically engineered trees with increased wood acetylation may be beneficial for other uses of wood, such as lumber and furniture.

## 12. Perspective

Genetic and biochemical studies in the past decade have revealed that members of the TBL family are *O*-acetyltransferases catalyzing *O*-acetyl transfer onto various plant cell wall polysaccharides. TBL members appear to be recruited to acetylate pectins as early as in charophyte green algae, and during the evolution of land plants, they underwent expansion via gene duplication and functionally diversified to act on additional cell wall polysaccharides. It will be important to find out how TBLs evolved from their ancestral genes to acetylate diverse plant cell wall polysaccharides, i.e., what structural changes TBLs underwent to enable them to accommodate different cell wall polysaccharides as acceptors. Among the 46 members of the *Arabidopsis* TBL family, 29 of them have been characterized and the functions of the remaining ones await elucidation. In addition, functional dissection of the exact roles of two other groups of proteins, RWAs and AXY9, involved in cell wall polysaccharide *O*-acetylation will provide mechanistic insights into the *O*-acetylation machinery of plant cell wall polysaccharides. Furthermore, although the *O*-acetylation of xylan has been shown to be critical for cellulose–xylan interaction, it has yet to be investigated whether and how the *O*-acetylation of xyloglucan, mannan, and pectins modulates cell wall structure. With the promising findings that transgenic plants with reduced cell wall acetylation show increased yields of fermentable sugars, the next important step is to engineer biofuel crops with reduced cell wall acetylation at a level that plants could tolerate, without adverse impacts on cell wall structure and plant growth, in order to maximize the benefits for biofuel production.
